# Boots for the Belgian Wounded

**Published:** 1914-12-19

**Authors:** J. J. Webb

**Affiliations:** Secretary. General Hospital, Tunbridge Wells


*To the Editor of* The Hospital.

### Boots for the Belgian Wounded.

*To the Editor of* The Hospital.

Sir,—I beg to acknowledge the safe arrival of four pairs of new boots for the wounded Belgian soldiers here who were without them.

They have been tried on and all found to be an excellent fit. The men are very grateful for them, and desire me to offer their heartfelt thanks for your kindness.

You would like to have some record of the wearers of the boots. Therefore I am sending you the following particulars :—

Gabriel de Maare, 4th Lancers, No. 14,972. Wounded at Alost. Bullet wounds in knee and temple.

Alphonse Melis, 8th Reg. de Ligne, No. 57,728. Wounded at Termonde. Bullet wound of arm.

Honoré Liebaert, 2nd Reg. de Ligne, No. 59,374. Wounded at Ghent. Bullet wound of arm.

Octave de Ryck, 1st Reg. de Carabineers, No. 1,207. Wounded at Termonde. Bullet wound of arm.

I am, Sir, yours faithfully,

J. J. Webb, Secretary.

General Hospital, Tunbridge Wells,
December 10, 1914.

